# The Impact of Pituitary Blockage with GnRH Antagonist and Gonadotrophin Stimulation Length on The Outcome of ICSI Cycles in Women Older than 36 Years

**Published:** 2014-07-08

**Authors:** Rosane Santana, Amanda Souza Setti, Luiz Guilherme Maldonado, Fernanda Montenegro Valente, Carla Iaconelli, Assumpto Iaconelli Jr, Edson Borges Jr

**Affiliations:** 1 Fertility-Assisted Fertilization Center, Av. Brigadeiro Luis Antônio, São Paulo, SP, Brazil; 2 Sapientiae Institute-Educational and Research Center in Assisted Reproduction, Rua Vieira Maciel, São Paulo, SP, Brazil

**Keywords:** Gonadotrophins, Implantation, Intracytoplasmic Sperm Injection

## Abstract

**Background:**

The objective of this retrospective cohort study was to evaluate whether the length of pituitary blockage with gonadotrophin-releasing hormone (GnRH) antagonists or the stimulation period influence intracytoplasmic sperm injection (ICSI) outcomes in patients older than 36 years of age.

**Materials and Methods:**

In this retrospective study, a total of 138 couples with maternal age >36 years undergoing ICSI with an antagonist protocol were included. The influences of stimulation and suppression length on the response to ovarian stimulation and ICSI outcomes were investigated. Receiver operating characteristic curve (ROC) analysis was performed to assess the predictive value of the stimulation period for achievement of implantation and pregnancy.

**Results:**

The gonadotrophin stimulation length negatively influenced the implantation rate (RC: -4.200; p=0.023). The area under ROC curve (AUC) could distinguish between women with positive and negative implantation (AUC: 0.611; CI: 0.546-0.673) and pregnancy (AUC: 0.593; CI: 0.528-0.656). The threshold value demonstrated a high negative predictive value on likelihood of implantation (p=0.0032, 90% sensitivity) and pregnancy (p=0.0147, 87.1% sensitivity) when patients underwent more than 10 days of stimulation.

**Conclusion:**

The stimulation period negatively influences the implantation rate in women older than 36 years. A stimulation interval greater than 10 days is associated with a negative predictive value for the chance of implantation and pregnancy.

## Introduction

The use of gonadotrophin-releasing hormone (GnRH) antagonists arose as a practical and less expensive alternative to the use of GnRH agonists for treating pituitary blockage. These compounds induce the internalisation and subcellular translocation of the GnRH receptor to the cell nucleus (1) and down regulate the expression of the GnRH receptor (2), provoking a rapid suppression of follicle stimulating hormone (FSH) and luteinizing hormone (LH) secretion (3, 4).

Two multiple-dose antagonist regimens are commonly used. The fixed protocol begins administration of the antagonist on day 6 of stimulation (3), while the flexible pituitary suppression protocol initiates treatment when a leading follicle with a mean diameter >14 mm is observed (5). The two protocols do not show a difference in preventing the incidence of LH rise (6).

In normal responders, the use of antagonists appears to have many advantages, including the avoidance of hypoestrogenic side effects caused by prolonged pituitary blockage (7) as well as lower gonadotrophin requirements, reduced cost and less time between cycles, as compared to GnRH agonist protocols (8). Moreover, the use of antagonists diminishes the risk of ovarian hyper stimulation syndrome (OHSS) (9-12).

Many different protocols have been used to improve pregnancy rates (PRs) in poor responders, including the use of GnRH antagonists such as cetrorelix (13, 14). The antagonist schedule is attractive as a treatment for poor responders because initiation occurs after the commencement of gonadotrophin stimulation in these patients and treatment with GnRH antagonists may have minimal impact on early follicular recruitment (14). However, the true impact of GnRH antagonist protocols in poor responders remains unclear. Some studies have shown that these treatments may improve the ovarian response and the number of retrieved oocytes (15, 16), while other data do not support these findings (14, 17-20).

Advanced age is one of the primary predictive factors associated with poor ovarian response (21). Advanced maternal age frequently correlates with cycle cancellation, a reduction in the number of embryos for transfer and decreased pregnancy rates.

No data evaluating whether the length of pituitary blockage with GnRH antagonist or the stimulation period influence intracytoplasmic sperm injection (ICSI) outcomes in patients older than 36 years, who are likely poor responders, are currently available. Therefore, the aim of this study was to collect these data.

## Materials and Methods

### Patient selection

We retrospectively evaluated cycles performed from January to December 2011 in a private assisted fertilization center located in Brazil. Inclusion criteria were: couples with maternal age >36 years undergoing ICSI cycles using an antagonist protocol, primary infertility, patients with regular menstrual cycles, FSH levels <10 IU/L measured at cycle day third, and normal uterine ultrasounds. Exclusion criteria were: women with severe endometriosis (stage III and IV) and azoospermic males. Of 735 cycles, 138 were considered for inclusion in this study.

Written informed consent, in which patients agreed to share the outcomes of their cycles for research purposes, was obtained from all patients, and the study was approved by the Local Institutional Review Board.

### Ovarian stimulation and oocyte retrieval

The patients started recombinant FSH (rFSH) treatment (Gonal-F®, Serono, Geneve, Switzerland) daily from the third day of their menstrual cycles. The first ultrasound control and the estradiol (E2) plasma dosage tests were performed at the seventh cycle day. Depending on the response of each patient, controlled by ultrasound monitoring of the follicles size, the dose of rFSH was adjusted. GnRH antagonist (cetrorelix acetate, Cetrotide; Serono, Geneva, Switzerland) was administered when the dominant follicle was 14 mm in mean diameter. Oocyte retrieval was performed 35 hours after the administration of recombinant human chorionic gonadotrophin (rhCG; Ovidrel™, Serono, Geneve, Switzerland) through transvaginal ultrasonography.

### Preparation of oocytes, intracytoplasmic sperm injection and embryo culture

Retrieved oocytes were maintained in culture media (Global® for fertilization, LifeGlobal, Connecticut, USA) supplemented with 10% protein supplement (LGPS; LifeGlobal, Connecticut, USA) and covered with paraffin oil (Paraffin oil P.G.; LifeGlobal, Connecticut, USA) for two to three hours before removal of cumulus cells.

ICSI was performed in a micro-injection dish prepared with 4 μL droplets of buffered medium (Global® w/HEPES, LifeGlobal, Connecticut, USA) and covered with paraffin oil on a heated stage at 37.0 ± 0.5˚C of an inverted microscope.

Approximately, 16 hours after ICSI, fertilisation was confirmed by the presence of two pronuclei and the extrusion of the second polar body. Embryos were maintained in a 50 μL drop of culture medium (Global®, LifeGlobal, Connecticut, USA) supplemented with 10% protein supplement covered with paraffin oil in a humidified atmosphere under 6% CO2 at 37˚C for three days. High-quality embryos were defined as those showing 8-10 cells on the third day of development, less than 15% fragmentation, symmetric blastomeres, absence of multinucleation and absence of zona pellucida dysmorphisms.

All embryo transfers were performed by the same gynaecologist, on day third of embryo development, using a soft catheter with transabdominal ultrasound guidance.

### Clinical follow-up

The luteal phase supplementation was started one day after oocyte retrieval according to the serum E2 levels of each patient on the day of hCG administration (ovulation trigger), with a vaginal administration of 600 mg daily of micronized progesterone (P4) and 200 μg of transdermal E2 for patients with E2<2.000 pg/ml. P4 alone was administered to patients with elevated E2 serum levels. P4 supplementation was continued until 12 weeks of gestation in the presence of a positive hCG test.

A pregnancy test was performed 12 days after embryo transfer, a positive pregnancy test was considered to define a biochemical pregnancy. All women with a positive test were given a transvaginal ultrasound scan 2 weeks after the positive test. A clinical pregnancy was diagnosed when the foetal heartbeat was detected. Clinical pregnancy rates were calculated per transfer. Miscarriage was defined as spontaneous abortion before 20 weeks of gestation.

### Statistical analysis

All results are expressed as the mean ± standard deviation for numeric variables, while proportions (%) are used for categorical variables. Mean values were compared by Student’s t test or Mann-Whitney test, while percentages were compared by the chi-squared or Fisher exact test, only when expected frequency was five or fewer. Regression analyses models were used to evaluate the influence of stimulation and suppression length on the outcomes. For numerical outcomes such as the number of follicles, retrieved oocytes, oocyte recovery rate, metaphase II (MII) oocyte rate and fertilisation rate, linear regressions were used. For categorical variables such as implantation rate, pregnancy rate, miscarriage rate and embryo transfer rate, logistic regressions were used.

Additionally, we divided the patients into two groups, ≤4 and >4 days, according to the length of antagonist suppression in order to investigate whether or not prolonging the suppression phase would influence ovarian response to COS and/or ICSI outcomes.

Receiver operating characteristic (ROC) curve analysis was performed to assess the predictive value of the stimulation period on implantation and pregnancy achievement rates.

The results of linear regressions are expressed as regression coefficients (RC) whereas the results of logistic regressions are expressed as odds ratios (OR) with 95% confidence intervals (CI). The ROC curve results are expressed as area under curve (AUC) with 95% CI. The results were considered to be significant at the 5% critical level (p<0.05). Data analyses were carried out using the Minitab® and MedCalc® statistical programs.

## Results

Characteristics of the cycles are listed in table 1. The lengths of pituitary suppression and gonadotrophin stimulation were 4.2 ± 1.1 and 9.7 ± 1.3 days, respectively. The influence of these treatment periods on ICSI outcomes is shown in table 2. The suppression and stimulation lengths had no influence on the number of follicles, the number of oocytes, oocyte recovery rate, MII oocyte rate, fertilisation rate or embryo transfer rate.

The length of antagonist suppression treatment displayed no influence on the implantation rate, pregnancy rate or miscarriage rate. However, the gonadotrophin stimulation negatively correlated with the implantation rate and tended to predict diminished odds of pregnancy and increased odds of miscarriage (Table 2).

**Table 1:** General characteristics of the cycles and ICSI outcomes


Variable	Value

**Mean female age (Y)**	40.2 ± 2.4
**Mean total dose of FSH administered (IU)**	2620.8 ± 626.9
**Mean number of follicles**	11.0 ± 9.1
**Mean number of oocytes**	7.6 ± 6.1
**Oocyte recovery rate (%)**	1052/1517 (69.8)
**MII oocyte rate (%)**	794/1052 (75.5)
**Fertilisation rate (%)**	511/793 (64.4)
**Mean number of transferred embryos**	2.4 ± 1.3
**Implantation rate/transferred cycle (%)**	31/293 (10.6)
**Pregnancy rate/transferred cycle (%)**	31/121 (25.6)
**Miscarriage rate (%)**	10/31 (32.2)


MII; Metaphase II and ICSI; Intracytoplasmic sperm injection.

**Table 2:** Regression analyses results of the influence of suppression and stimulation lengths on ICSI outcomes


Outcome	Suppression length	Stimulation length

**Number of follicles**	RC: 0.7073, p=0.326	RC: 0.3203, p=0.413
**Number of oocytes**	RC: 0.5283, p=0.274	RC: 1.230, p=0.372
**Oocyte retrieval rate**	RC: 1.228, p=0.470	RC: 0.2286, p=0.504
**MII oocyte rate**	RC: 2.712, p=0.116	RC: 0.499, p=0.743
**Fertilisation rate**	RC: 1.703, p=0.251	RC: -1.345, p=0.299
**Embryo transfer rate**	OR: 1.67, CI: 1.02-2.74, p=0.360	OR: 1.40, CI: 0.98-2.00, p=0.162
**Implantation rate**	RC: -2.033, p=0.315	RC: -4.200, p=0.023
**Pregnancy rate**	OR: 0.89, CI: 0.60-1.31, p=0.555	OR: 0.69, CI: 0.48-1.01, p=0.051
**Miscarriage rate**	OR: 1.20, CI: 0.52-2.77, p=0.658	OR: 2.04, CI: 0.81-5.11, p=0.088


MII; Metaphase II, RC; Regression coefficient, OR; Odds ratio, CI; Confidence interval and ICSI; Intracytoplasmic sperm injection.

No significant differences were observed between the patients divided into groups treated with ≤4 and >4 days of suppression in the female age 40.0 ± 2.4 vs. 40.4 ± 2.4, p=0.4397; number of follicles 11.3 ± 8.3 vs. 10.8 ± 9.7, p=0.4327; number of oocytes 8.1 ± 6.3 vs. 7.3 ± 6.5, p=0.4311; oocyte retrieval rate 71.8% vs. 68.0, p=0.3168; MII oocyte rate 75.1% vs. 76.2; fertilisation 61.9% vs. 67.6, p=0.3828; implantation 9.5% vs. 11.8, p=0.8674; pregnancy 27.3% vs. 24.2, p=0.7038 or miscarriage rates 40.0% vs. 25.0, p=0.4578.

**Fig 1:**
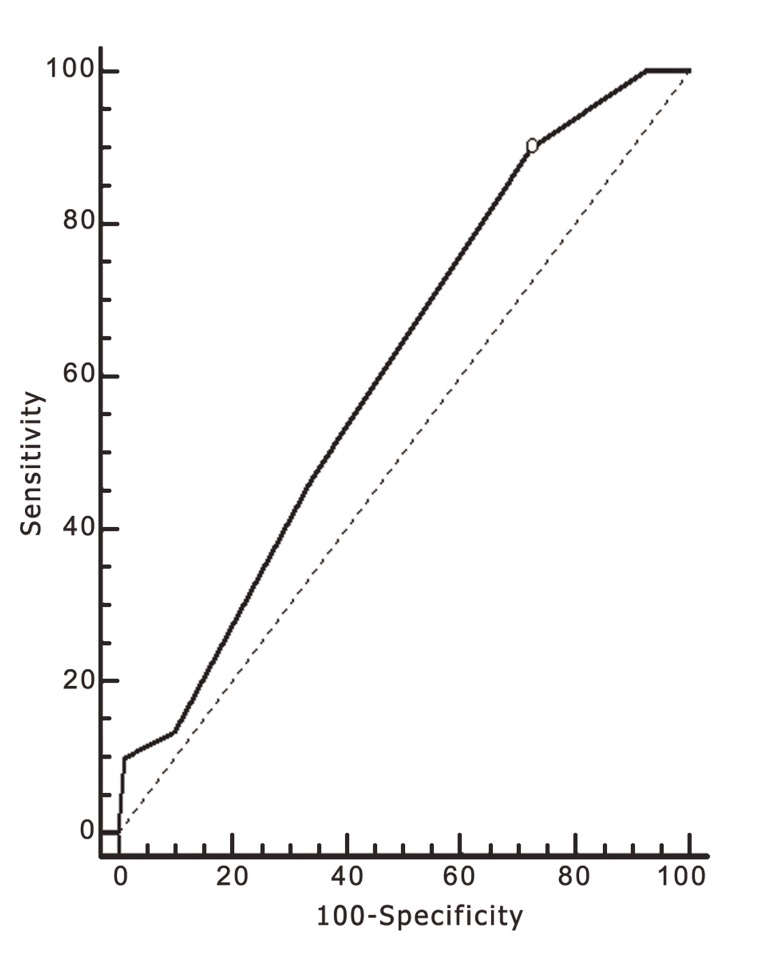
ROC curve for AUC period of stimulation with dependent parameter, the achievement of implantation.

**Fig 2:**
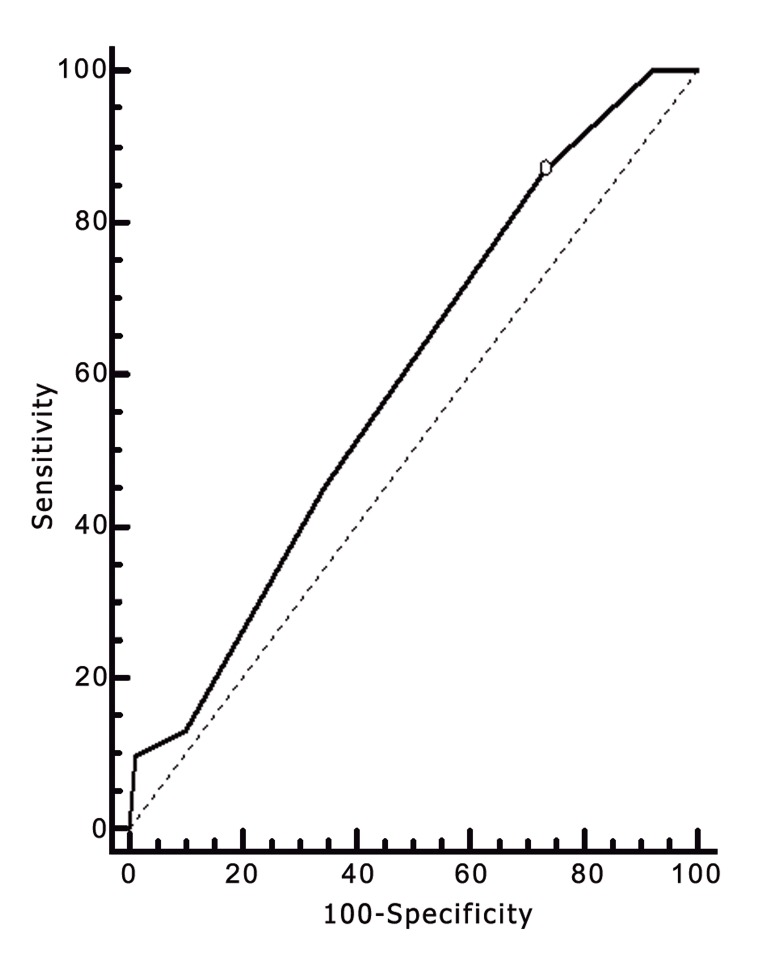
ROC curve for AUC period of stimulation with dependent parameter, the achievement of pregnancy.

A ROC curve analysis was performed to assess the predictive value of the period of stimulation on the achievement of implantation and pregnancy. The calculated AUC was sufficient to distinguish between women with positive and negative implantation (Fig 1, AUC: 0,611; CI: 0,546-0,673) and pregnancy (Fig 2, AUC: 0, 593; CI: 0,528-0,656).

The threshold value showed a high negative correlation with the chance of implantation (p=0.0032, 90% sensitivity) and pregnancy (p=0.0147, 87.1% sensitivity) for patients treated with more than 10 days of stimulation. In the group of patients who underwent 11 days of stimulation (n=22), 3 of the 4 pregnancies achieved (18.1%) resulted in pregnancy losses (75.0%). Furthermore, in the group of patients who underwent 12-13 days of stimulation (n=11), no implantations or pregnancies were achieved.

## Discussion

Increased daily dosage of gonadotrophins, in combination with different pituitary blockage protocols, has been used as the major approach to enhance follicular recruitment in women with advanced age or poor ovarian response. However, it is unclear whether this treatment influences ovarian response.

While most studies of gonadotrophin administration focus on the total dose of drugs administered, this study focused on the relationship between the length of gonadotrophin stimulation and ICSI outcome. We observed that stimulation length negatively correlates with implantation rate and tends to predict diminished odds of pregnancy and increased odds of miscarriage.

In an attempt to establish a specific threshold stimulation phase length, beyond which pregnancies and implantations failed to occur, we performed ROC curve analyses and observed that periods of greater than 10 days of stimulation showed a high negative predictive value on the chance of implantation and pregnancy. However, it is important to mention that, to be considered good, an AUC should be >0.8, and the AUCs obtained in this study (0,611 for implantation and 0,593 for pregnancy) are therefore considered poor (22). We also observed that stimulation periods longer than 11 days resulted in no implantations or pregnancies.

Alport et al. (23) showed that a short or long ovarian stimulation phase length is associated with a suboptimal number of follicles developing serum estradiol concentrations and number of oocytes retrieved in couples undergoing *in vitro* fertilization (IVF). However, in contrast to our findings, the authors observed that the length of the stimulation phase does not predict embryo development or pregnancy outcomes.

The length of follicular stimulation is determined by the amount of time required for the ovary to produce a minimum number of follicles with a certain mean diameter. Kerin et al. (24) suggested that the ideal stimulation protocol should promote the development of at least three follicles with at least 17 mm in diameter each, which would yield at least two embryos available for transfer.

The stimulation phase of IVF cycles appears to be an independent predictor of implantation and pregnancy outcome. Our data suggest that in women with advanced age, the ICSI outcome depends not only on the development of size-appropriate follicles, but also on the speed at which the ovaries develop these follicles.

Martin et al. (25) found no significant difference in pregnancy rates between women who were stimulated for <9 days, 10-11 days or >12 days. However, in an attempt to correct their groups for equality of response, their study considered only patients who yielded between 10 and 12 oocytes, and the pregnancy and implantation rates were significantly higher in the patients with shorter stimulation phases. An important difference between our studies may account for the disparate result. Our study included only patients with advanced maternal age (mean female age of 40.2 years), while Martin’s study involved no age selection (mean female age of 35.9 years).

Increased utilisation of gonadotrophins has been shown to associate with poor pregnancy rates following IVF (26). Although the exact mechanism of detriment attributable to excess gonadotrophins is unclear, adverse influences on the granulosa cells of the developing follicle, the oocyte, the embryo, the endometrium and the metabolic milieu have been described (27-29).

Pal et al. (30), demonstrated that excessive administration of gonadotrophins during IVF allows a higher percentage of cycles to proceed to egg retrieval, albeit at the expense of lowering clinical pregnancy and live birth rates. Their data also suggest that high doses of gonadotrophins may translate into higher rates of spontaneous miscarriage.

It has been suggested that tailoring the start day of GnRH antagonist administration to a leading follicle size of 14-15 mm (flexible protocol), instead of utilising the traditional fixed protocol, could improve ovarian stimulation (31). However, the available data show that pregnancy rates tend to be lower when using the flexible protocol. Some researchers have shown that the mean day for initiation of the GnRH antagonist was day 7 of stimulation in the flexible protocol, while antagonist treatment is started on day 6 of stimulation in the fixed protocol. It is possible that this discrepancy explains the higher effectiveness of the fixed protocol (32-34).

We considered that the mean day of the commencement of GnRH antagonists used in the fixed protocol is day 6 of stimulation, while the flexible protocol begins on day 7. Additionally, the most common average length of stimulation is 10 days. Based on these data, patients enrolled in this study were divided into two groups: those who underwent 4 or less days of GnRH antagonist treatment, and those who underwent more than 4 days of treatment.

Although previous studies have shown that the two multiple-dose protocols of antagonists (flexible and fixed), and consequently the different lengths of pituitary blockage, interfere with ICSI outcomes (32-34), this study did not confirm these previous findings. One possible explanation for this difference is that our study included a homogenous group of patients, overcoming the liability of most studies concerning the use of antagonists in more heterogeneous populations.

In addition, it is known that a luteinizing hormone surge is associated with decreased probability of pregnancy because ovulation prior to oocyte retrieval may occur, and the LH surge may result in premature secretory transformation of the endometrium (33).

In comparative trials, the incidence of LH surge and premature LH rise was higher in the antagonist group compared with the agonist group (35), and LH rise seems to occur before the initiation of antagonist treatment (11, 36). Therefore, the use of the flexible protocol, which initiates GnRH antagonist treatment later than in the fixed protocol, could result in worse PRs because of a premature LH rise and corresponding negative impact on the endometrium.

On the other hand, there is evidence that antagonist treatment induces profound suppression of endogenous LH and that although this treatment is not required for follicle growth, it is important for follicle dynamics in the late follicular phase (37-39). Thus, the lack of LH and the impact of a prolonged length of pituitary suppression could be quite significant in the fixed protocol.

## Conclusion

The length of treatment with the GnRH antagonist has no influence on the outcome of ICSI in women older than 36 years. However, our data contribute to the consensus in the literature suggesting the detrimental prognostic impact of increased gonadotrophin requirements during IVF cycles. The threshold value showed a high negative predictive value on the chance of implantation and pregnancy with more than 10 days of stimulation. Therefore, we suggest that mild ovarian stimulation protocols should be used, even in patients with advanced maternal age.
